# Living beyond the limitation: Rehabilitation, life and productivity of individuals with schizophrenia in South‐West Nigeria

**DOI:** 10.1111/hex.13139

**Published:** 2021-01-11

**Authors:** Oyeyemi Olajumoke Oyelade, Nokuthula Gloria Nkosi‐Mafutha

**Affiliations:** ^1^ Department of Nursing Education School of Therapeutic Sciences University of the Witwatersrand Parktown Johannesburg South Africa; ^2^ Department of Nursing Science, Faculty of Basic Medical Sciences Obafemi Awolowo University Ile-Ife Osun-state Nigeria

**Keywords:** mental health service users, productivity, rehabilitation, schizophrenia

## Abstract

**Introduction:**

Schizophrenia, the most chronic and stigmatized form of mental illness, can be described as a brain disorder that affects an individual's cognition. Individuals with schizophrenia exhibit socially unacceptable symptoms that affect their psychosocial lives. They suffer from reduced productivity due to the debilitating effect of the illness, and the negative symptoms impede their employability; such symptoms and effects aggravate the stigma around mental illness. However, when rehabilitation is successfully achieved, so is productivity, and this decreases the associated stigma. Thus, this study describes the rehabilitation experiences and productivity of individuals with schizophrenia in South‐West Nigeria.

**Methods and analysis:**

A descriptive qualitative approach with semi‐structured interviews was used to gather information from mental health service users. The discharged users in this study received in‐hospital or outpatient rehabilitation care at four outpatient units within two specialist mental health‐care facilities in South‐West Nigeria. These facilities offer vocational training and rehabilitation services for individuals with schizophrenia. Twenty‐nine mental health service users were interviewed. The data from the interviews were independently analysed by two researchers through a content analysis approach using NVIVO version 11. The researchers compared the results of the analysis and reached an agreement on the conclusion.

**Findings and recommendations:**

The rehabilitation services availed by patients in the research setting are of three types. Some attend occupational rehabilitation to learn a trade; they depend on professionals for the choice of skill but at a cost that is not affordable to many. Some stay in rehabilitation units linked to the hospital, rendering their services at a cost, and their living expenses and skill acquisition processes are based on the remuneration they get from the services rendered to the institution. Others depend on their family members' efforts to afford rehabilitation services but set up on job by family or employed in family business. The mental health service users in this study who offered their services to the institutions were able to make informed decisions and showed better performance with their chosen occupation than those who depended on their family or health professionals for the choice of rehabilitation service or vocational career. Therefore, this study concludes that prioritizing mental health facility users' preferences in terms of productive activities (sales, services, vocation) or rehabilitation goals should be encouraged.

## 1 BACKGROUND

Schizophrenia constitutes 1% of the total global population^1^ and 7% of the global burden of mental illnesses^2^; however, there is a dearth of resources to estimate its prevalence in Africa. The rate of global increase in mental illnesses is considered significant since it has the highest rate of disabling effects (regarding low or no productivity) on individuals.^3^, ^4^ Schizophrenia has associated behavioural features that result in psychosocial challenges,^5^ and it occurs at the stage of role identity development, that is 18‐25 years among men and 25‐35 years among women.^1^ Individuals with schizophrenia lack motivation, face a reduced drive and impeded functionality and productivity,^6^ with the latter affecting the family and national economy.^7^ This is corroborated by global statistics—individuals with schizophrenia are at 24 million,^8^ and health‐care expenditure for schizophrenia is at 7%‐12% of the gross national product (GNP) in each country.^7^ In addition, individuals with schizophrenia are more prone to diabetes mellitus and cardiovascular disease, which are leading causes of long‐term disability.^9^, ^10^ Two scholars have previously attributed low productivity to endocrine and cardiovascular disorders, which is aggravated when compounded by mental illness.^11^, ^12^

Life expectancy of schizophrenic individuals is usually 25 years lesser than the norm due to inactivity and associated disorders.^10^ This strengthens the need for a proper rehabilitation plan before re‐integrating individuals into the community.^13^ It is important to note that the pursuit of rehabilitation for those living with schizophrenia commences before patients are discharged from the hospital and involves different therapies, including pharmacotherapy, family therapy, group therapy and occupational therapy.^14^ The ideal step for instituting these therapies should be through shared decision‐making processes.^15^ However, the application of these processes varies for each therapy. Pharmacotherapy involves medication through the active participation of the patients on preferred choice and dosage^16^; family therapy focuses on family and societal support through acceptance and empowerment^17^; group therapy entails therapeutic use of recovered and well‐rehabilitated individuals as role models for others with mental illnesses^18^; and occupational therapy entails the re‐training of previous social and vocational skills, which enables communications, relationship management and trade.^19^

The benefits of rehabilitation are abound in the literature. It reduces disability, improves the quality of life and increases life expectancy among schizophrenic individuals.^20^ While it does ensure productivity, it has been found that rehabilitation that entrenches independent living is best suited for optimal functioning.^21^, ^22^ Studies have shown that rehabilitation reduces the community stigma around mental illness^23^ and exerts positive effects on the life and environment of individuals with schizophrenia.^21^, ^22^, ^23^ Further, depression and suicide that arise from inactivity and hopelessness can be prevented by productivity.^24^ However, there are limited studies on the rehabilitation and productivity of individuals with schizophrenia and the burden on low‐ and middle‐income countries.^25^, ^26^ Therefore, this study was focused on assessing and describing the experiences, rehabilitation and productivity of schizophrenic individuals in South‐West Nigeria and suggests possible ways of improvement.

## 2 RESEARCH METHODS

The experiences of individuals living with schizophrenia, their rehabilitation and levels of activity and productivity were studied, including their expectations of the rehabilitation exercise, the perceived benefits, changes since they began engaging in rehabilitation, the activities they engage in, their productivity levels, their concerns about rehabilitation and suggestions for improvement. A descriptive study design was adopted with a qualitative approach to data collection, specifically using semi‐structured interviews. Purposive sampling was used to select participants who were mental health service users living with schizophrenia, discharged from hospital but receiving in‐hospital or outpatient rehabilitation care.

The interviews were conducted in April–September 2019, with each interview lasting 45‐90 minutes. The researcher conducted about one to three interview sessions with each participant. The purposive sampling of mental health service users with schizophrenia was performed through record reviews and inputs from assistants of health professionals. The research setting constituted 62 patients accessing rehabilitation services, and 50 were eligible for the interview; however, 21 refused to give consent, while some withdrew their consent at the point of interview. In total, 29 mental health service users who gave their consent were interviewed. Table 1 depicts the demographic description of the participants.

**TABLE 1:** Socio‐demographic characteristic of participants

S/N	Unit	Rehabilitation experience (years)	Vocation before illness	Vocation after illness	Dependency
1	Psychogeriatric Unit	18	Business (Major distributor of rubber)	Daily house cleaning	Accompanied by relative
2	Psychogeriatric Unit	20	Retired Typist	Daily house cleaning and cooking	Accompanied by relative
3	Psychogeriatric Unit	2	Food Vendor	Daily house cleaning	Accompanied by relative
4	Psychogeriatric Unit	5	Caterer	Nanny (Baby‐sitting)	Accompanied by relative
5	Psychogeriatric Unit	20	Petty Trader	Daily house cleaning and cooking	Alone, not accompanied
6	Psychogeriatric Unit	6	Petty Trader	Little house care with paid house help	Accompanied by relative
7	Psychogeriatric Unit	7	Fashion Designer	Fashion Designer	Alone, not accompanied
8	Rehabilitation Unit	10	Native Doctor	Self‐care(grooming)	In‐house, no relatives
9	Rehabilitation Unit	10	Primary School Certificate Holder	Hospital Cleaner	In‐house, no relatives
10	Rehabilitation Unit	9	Mechanic	Hospital Cleaner	In‐house, no relatives
11	Rehabilitation Unit	10	Carpenter	Self‐care	In‐house, no relatives
12	Rehabilitation Unit	30	Carpenter	Hairdressing and In‐house Petty Trade	In‐house, no relatives
13	Rehabilitation Unit	41	High School Certificate Holder	Hospital Cleaner	In‐house,no relatives
14	Rehabilitation Unit (Hope Villa)	19	High School Certificate Holder	Sells Recharge Card	Occupant of a room in the hospital Private residence
15	Rehabilitation Unit (Hope villa)	4	Retired Teacher	Hospital stock shop keeper	Occupant of a room in the hospital Private residence
16	Rehabilitation Unit	5	Housewife (Home‐manager)	Hospital Cleaner	In‐house, no relatives
17	Rehabilitation Unit	6	Junior School Certificate (JSSCE)Holder	Hospital staff Vehicle cleaner	In‐house,no relatives
18	Occupational Therapy Unit	20	University Student	Tailoring apprentice	Alone, not accompanied
19	Occupational Therapy Unit	15	University Graduate	Hairdressing	Alone, not accompanied
20	Occupational Therapy Unit	29	House‐help	Leisure walk	Alone, not accompanied
21	Occupational Therapy Unit	32	University Graduate(Public administration)	Hairdressing apprentice	Alone, not accompanied
22	Occupational Therapy Unit	22	High School certificate Holder	Wool work(Knitting) apprentice	Alone, not accompanied
23	Occupational Therapy Unit	37	Real Estate Manager	Learning Computer	Alone, not accompanied
24	Occupational Therapy Unit	2	Funnel Taker/Office Assistant	Learning Computer	Alone, not accompanied
25	Occupational Therapy Unit	32	Primary school Certificate Holder	Hairdressing apprentice	Alone, not accompanied
26	Occupational Therapy Unit	9	Graduate(Business Administration)	Tailoring apprentice	Alone, not accompanied
27	Occupational Therapy Unit	10	Quantity Surveyor	Different units‐from tie and dye to shoemaking, to tailoring, now computer	Alone, not accompanied
28	Community service centre	2	University Graduate	Shop keeper	Alone, not accompanied
29	Community Service Centre	1	Farmer	Farmer	Alone, not accompanied

### 2.1 Research setting

This study was conducted in South‐West Nigeria, which is where humanitarian psychiatric care first took off.^27^ The region houses two institutions and has the largest capacity for mental health‐care services in Nigeria. These institutions offer comprehensive mental health services and have facilities for rehabilitation and vocational training of individuals with mental illness and schizophrenia. They are located approximately 120 km from each other; the first was established 37 years before the second and has more space and facilities.

The first institution has three units that engage in vocational and rehabilitation activities: the psychogeriatric unit (a strictly outpatient unit where older adults, specifically 46 years or older, are seen); rehabilitation wards/units (where discharged patients who cannot go home immediately or have no place to go are engaged); and the outreach clinic (distant from the main hospital and primarily serves as a rural community psychiatry service centre). Unlike the outreach clinic, the rehabilitation wards are within the hospital and have nurses as home matrons. Notably, several mental health service users who reside in rehabilitation units work as hospital cleaners and receive monthly stipends, using which they pay accommodation fees and buy medication. Some of the residents of these rehabilitation wards are retired government employees on pension. The rehabilitation units have private lodges called Hope Villas for residents who prefer, and can afford, private facilities.

The second institution has only one unit that handles the rehabilitation of individuals with schizophrenia—the occupational therapy unit. Mental health service users come to this unit daily to engage in different rehabilitation services, from learning vocational skills (tailoring, hairdressing, shoemaking, bead‐making, wool works) to behavioural habit training and psychosocial therapies (relationship and anger management).

### 2.2 Sampling and procedure for data collection

Participants were purposively selected, and the prospective participants' files were sorted with the assistance of record officers before being reviewed by the investigator and nurses on duty. Inclusion criteria were that the individual had to have a diagnosis of schizophrenia and been mentally stable for not less than 3 months (research evidence shows that the risk of relapse is usually high from 1‐3 months of discharge^28^). Exclusion criteria were records of positive symptoms in the last 3 months (which can impede the interview process, such as flight of idea or slurred and incoherent speech), history of debilitating co‐morbidity (such as head injury, cerebral cancer, poorly managed diabetes with injuries or wounds), affective co‐morbidities (such as depression and mania) and individuals younger than 18 years. Those who satisfied the inclusion criteria were contacted and briefed about the study.

The eligible participants gave informed consent prior to their interview appointments. The interview sessions were held at each participant's preferred time, date and venue. Most of the participants were offered incentives (transport fare) at the close of the interview session; those who resided and were interviewed within the mental health facility were not given any transport fare but were thanked verbally.

### 2.3 Qualitative rigour

As previously stated, all the participants gave informed consent. To ensure the appropriateness, clarity and congruence of the study content with the set objectives and interview guide, the research was presented to a peer review committee as well as an expert review committee. In addition, the interview guide was reassessed post‐validation. The fieldwork was performed by the first investigator (OO), and the co‐researcher was periodically briefed at each stage of data collection. All the interview sessions, except one, were in English, and all of them were audio‐recorded with the participants' permission and transcribed verbatim. There was a member checking with the participants after data transcription. Back translation occurred for the entire script of the one interview session conducted in the Yoruba language. Two researchers coded the data until they reached a consensus on the emerging themes. These steps, along with the efforts described below, enhance the credibility and trustworthiness of the work.

### 2.4 Data analysis

Data analysis was performed using NVIVO 11 and the content analysis technique.^29^ The transcribed script was imported into the software, and nodes and sub‐nodes were developed. The nodes (primary node) presented the information pertaining to each particular question, while the sub‐nodes constituted information with similar meanings under each primary node (also regarded as categories). Similar categories were classified into themes, and the categorized data were reviewed for congruency with the corresponding principal nodes. Two independent researchers individually analysed the data before comparing the results and reaching a consensus. Four themes emerged from the analysis of participants' accounts, as described in the Results section. Table 2 presents the corresponding categories and themes.

**TABLE 2:** The themes and corresponding categories

Theme	Category
Rehabilitation approaches	Family/self‐initiated productivity Hospice techniques/services Alternative/traditional therapy
Perceptions	Hopeful vs positive changes Lack of insight vs wrong diagnosis Anger vs acceptance of fate
Expectations	Re‐integration to communal and social life Rehabilitation to the previous functioning Rehabilitation through gaining desired skills and not prescribed skills
Rehabilitation barriers	Structural barrier Psychosocial barrier Financial barrier

## 3 RESULTS

Table 1 presents the participant demographics. As reflected in the table, there were 17 male and 12 female participants of 20‐80 years of age and a mean of 53 years (Table 3 presents further details).

**TABLE 3:** Statistical summary of participants' ages

SN	Age	Frequency	Mean
1	20‐30	3	1542 ÷ 29 = 53.172~53
2	31‐40	4	
3	41‐50	4	
4	51‐60	7	
5	61‐70	9	
6	71‐80	2	
Total	1542	29	
	Mean	53	

### 3.1 Theme 1: Rehabilitation approaches

The first theme generated in the description of the participants' engagement was rehabilitation approaches.

This was expressed in three different perspectives which connotes, self and family initiative, hospice service and alternative/ traditional therapies.

In the description of self and family initiative, some of the participants reported that, although they were receiving training in rehabilitation units, they were living within their respective communities and considered themselves productive through family/self‐initiated efforts. Their declaration of family support is expressed in the statements that;

I stay with my mother in her house and co‐manage her shop with her … We sell provisions, and I have financial independence through the shared gains from the products we sell. (C15Ag)

I started a petty trade from capital I got from my children. I pay my house rent from it and do everything myself. They only come visiting at will, and they are always happy that I am not a burden to them. (C16Ag)

On the other hand, one participant said that she faces no productivity‐related challenges as far as her illness is concerned. Her response shows self‐initiative, as she was able to understand her own capabilities and chose the activity she found the easiest:

I tried going into sales and supply, but that made me anxious. Later, I tried fashion designing, with which I am coping well. Besides giving me financial independence, I do it at my convenience. (CR12Ao)

Another group of participants reported another approach to rehabilitation which connotes hospital‐based/stay training, hospital‐based paid services, plus self‐services within the hospital (hospice techniques). In their description, some said that they were provided job opportunities by the institutions and that the salary they receive from the hospital is sufficient for self‐sustenance. The job description reported by one of the participants is as follows:

We are employees of the hospital and on payroll … our employment is not from the government, but the hospital pays us. We clean and sweep every morning. (C8Ar)

Similarly, another participant talked about the supportive nature, salary and use of the job the institution provided them:

We serve as the hospital cleaners. We are trained to clean; we clean … It is this salary that we use to maintain ourselves. It's 200 naira per day (0.5 USD). (C13Ar)

Two other participants stated that the scope of their work went beyond cleaning offices; they engage in some menial jobs for additional self‐support alongside their institutional jobs. They described themselves as independent and able to meet their needs without having to depend on anybody as they said;

We live here and clean offices and the toilets on the wards. In addition, I go from bed to bed to shave the hair of patients and also sell snacks. These help me to buy my medication and pay for my accommodation. I don't depend on anybody. (C12Ar)

Besides the cleaning duty, I also sell sachet water using the hospital fridge. I also sell cell‐phone recharge cards to the nurses and the doctors. (C1Av)

Besides job opportunities, hospice services also provide training. The participants reported going to the occupational therapy (OT) unit of the hospital for training on self‐grooming, cooking and baking, hair dressing, computer software, shoemaking and sewing. The training results, as per participant's productivity and perceived benefits, were as follows:

I think they have been helping me so much in the OT that I have been attending … the kitchen rehabilitation … the salon. The catering section. I have passed through all of them. I think the way they are helping us is that they have been equipping me to be able to pick a skill. (C6L)

I am into tailoring. I have learnt measurement, I have learnt to spread, but it remains to sew. I am here because they felt it's best for me. (C1L)

I have learnt knitting, and I have mastered it and happy I did. What is left for me is to get materials and knitting machine. (C2L)

Apart from the self‐initiated efforts and hospice productivity techniques, some participants reported being taken to a traditional facility (alternative therapy) against their consent because their relatives considered it their family's preferred approach of rehabilitation and or a cheaper alternative. The participants reported this as unpalatable since they were confined, and any effort to liberate themselves led to violence. The participants' descriptions of the confinement and resultant effects were as follows:

My father took me to the house of an herbalist. Though I told him to bring me to the hospital, he said I should remain there. They tied me on the floor to a tree in the bush. I was so furious and tried to run away, but the herbalist arrested me. It was during that time that I injured someone, and the person died. (C12Ar)

Similarly, another participant stated the following:

This is my second time here. After the first time, they had no money to bring me back to this place, so they carried me to herbalist house because my people are poor, and they tied me down there doing many things. After that, I had a misunderstanding with my father during which I mistakenly killed him. (C13Ar)

Thus, the participants had varied experiences as far as their rehabilitation efforts are concerned.

### 3.2 Theme 2: Perception

The theme of perception emerged from the participants' view of rehabilitation exposure and the description of its impact on them. Some participants described rehabilitation as bringing hope, positive changes and improving their quality of life. They reported that their experiences help remove loneliness and boost human relations as well as enable them to see life as worth living, which they believe is vital for their productivity and effective living as they said;

When I was diagnosed of this illness, I had to stop school and had to stay at home doing nothing … not being active is worse than the illness. I felt like dying then … but with this rehabilitation that I am undergoing, I am now in order, doing well, now at OT. I am into knitting, so as I progress further … I no longer feel idle, and I know I will be well settled after completing the training. (C2L)

I have been gaining a lot because I am an introvert. So, I don't relate because I don't talk that much, but since I came in now, I have been able to come out of that attitude of being an introvert … I talk to people and relate well. No longer shy. I know I will go back fully to my work when I am very okay and can relate very well. (C8L)

However, some participants expressed perceptions of having been incorrectly diagnosed and showed a lack of insight into their diagnosis. These participants perceive their rehabilitation as unnecessary and having no basis. They believe they were not provided with sufficient/appropriate information to convince them of the illness. Further, they claimed to have been productive before being admitted to the hospice by health professionals. These participants are at the rehabilitation unit of the institutions and stated the following:

They said we are sick and want us to believe that we are sick, so we are here now receiving treatment because they said we are sick. I was at my duty post when co‐workers took me to a health centre, and it is the nurses at the health centre that brought me here. My family came for a while, and later they stopped coming. Now, they said we are doing rehabilitation. I believe they don't know what is wrong. (C11Ar)

I was referred from a private hospital, where I was diagnosed of malaria. The first time I came, the referral letter was sealed, and what l was told is that where I was referred to is a specialist hospital and that I should never open the letter but give it to the health professionals there. They told me I would be better managed. However, I am here because they want me here. (C1Av)

Some of the participants expressed anger and an acceptance of their fates. These individuals described themselves as unwanted and non‐functional due to the physical illness associated with their mental illness. They described their rehabilitation as having failed due to the belief that the illness grossly affected the extent to which they could be productive.

A participant shook her head, hissed and said,

I cannot even do anything, not even clean the house, just there sitting down. … I am just there useless. (C4Ag)

Another participant expressed anger with a gesture of displeasure as associated not only with the disability, but also with family neglect with the way she opened her hands, like she wanted to throw something away and said:

What can I do with the way I look … My mother does not want me again … she got rid of me… my mother brought me to the gate of the hospital and left me there … one of the doctor employed me as personal aid, but ejected me out of her house … since my mother rejected me, what can anyone not do to me? In such case, how is it possible for me to benefit from rehabilitation. (C3Ar)

Those with physical illnesses expressed feeling incapacitated during rehabilitation, while those who engaged in vocational training and services expressed a feeling of improvement. The others had a lack of insight about their illness and diagnosis.

### 3.3 Theme 3: Expectations

The participants' expectations were centred on what they wished to do with their lives based on what they expected from the rehabilitation programme. Some participants expressed that their utmost desire was to return to their respective homes and communities. These participants currently live within the hospital premises and guest house. Their occupational direction has been established, but they see their living within the hospital for rehabilitation as a form of bondage from which they desire freedom. Further, they recognize the fact that community life improves their chances of getting help and is best suited for them and expressed the following:

I wish to be back to the community. I love singing but can only do that well in the community. I have composed (songs), but I have nobody to help me. If I go back to the community, I will sing and get help … Like I composed a song which goes … There are many people in the bondage … they never wish to be in bondage. (C1Av)

I manage the stock shop owned by the hospital, but I have been living here for long. I am old now, and it is not good to stay somewhere and die there. The family may not even know the person has died. It will be very unpalatable and disheartening to live forever in a place outside your home. I desire they help me go back to the community, I can also do better business and make much sales at the community. (C7Av)

In contrast, the participants who live in their respective communities but visit the hospital for daily rehabilitation expressed their utmost desire to go back to the activities they had engaged in before the onset of their illness (rehabilitation to previous functioning). They believe they are doing well as far as rehabilitation is concerned and are determined to pursue their previous engagements. They believe that this will spare them the time required to venture into a new field. Excerpts from the participants' reports are given below:

what I want is for them to help me to achieve going back to my school and graduating. I feel I will do well going back to school rather than learning a trade and wish to complete my study and work as a professional in the discipline I studied. (C6L)

My aspiration in life is to become an administrative person, working for the government. I studied business administration at university and that's what I want to do. (C9L)

I was in school in a university outside the country when this illness started, but I was still studying. I had not gotten my degree; I still wish to study. (C7L)

Another expectation that was different from community re‐integration and previous patterns of functioning was the participants' rehabilitation through gaining desired skills and not prescribed ones. Participants stated that they sometimes desire the acquisition of new skills and not necessarily the ones they focused on before, but they desire to choose from the available options on their own and not have the professionals decide for them. They reported experiencing frustration with skill pursuit when it was the professional's choice and not a self‐driven one. An excerpt from a participant's recount is as follows:

I wanted computer (science) but was directed to tailoring by the HOD. There, I couldn't pass thread into the needle. I just wasted the three years. Later, the HOD agreed for me to learn computer … in this computer age, I would like to be a computer analyst. (C5L)

In summary, these participants expressed a desire for acceptance of their own views on skill acquisition and rehabilitation modality in general.

### 3.4 Theme 4: Rehabilitation barriers

This theme answers the questions on the participants' concerns about the programme and their suggestions for improvement. Some participants complained about a lack of infrastructure in the community, which impedes their functionality and rehabilitation (structural barrier). A participant recount goes thus:

I was brought back to the hospital by the social workers when the house I was to be discharged to was dilapidated. I do not even think anyone stays there anymore. (C7Av)

Another barrier was psychosocial in nature. Some participants revealed that there is fear and an actual expression of non‐acceptance by their family members. Social undesirability is a factor that limits their productive functioning in the community. Excerpts from a participant's statement is as follows:

I mistakenly killed my father. I have changed, but they do not want me at home. I was trained as vulcaniser here, and the institution got me equipment, but there is a challenge with going back home. There was a time I was discharged, but the family told them frankly that they can't accommodate me. If I decided to stay there by force, they could kill me. (C13Ar)

Further, some participants expressed financial difficulty in terms of setting up businesses and lack of financial capacity for medication and self‐upkeep. An excerpt from a participant's description is as follows:

My desire is that they should make effort to assist me to return home. But the means to using the medication is the utmost. If I go home and there is no job, getting money to buy medications for use will be difficult. So, if they can help me with getting the medications if I go home. Maybe I can sell petty things and start coming for check‐up. (C12Ar)

In short, these participants expressed their basic needs constituting barriers to rehabilitation.

## 4 DISCUSSION

This study revealed that individuals with schizophrenia are able to start small businesses, work in a formal setting and even learn new skills that will help them live and sustain a productive life. This is consistent with the finding that individuals with schizophrenia in paid jobs have boosted morale and increased quality of life.^30^ Although the participants in this study earn less than a dollar per day, they stated that the amount is sufficient to meet their basic needs.

In terms of perception, the participants held negative and positive beliefs about themselves. Their negative beliefs evoked a feeling of worthlessness, primarily resulting from the rejection they experienced from their family. Their positive beliefs arose from being actively engaged in a vocation. This is consistent with findings that inactivity can cause mortality among schizophrenic patients.^31^ Scholars have also reported that this feeling of worthlessness arises when individuals with schizophrenia experience abandonment by their family for any reason.^1^, ^32^, ^33^ Notably, some participants' perceptions were influenced by defective insights into their diagnoses. These participants had been hospitalized for about 30 years and in rehabilitation for 20 years. The general expectation was that they would have some level of insight or sense of identity from the rehabilitation services and pharmacotherapy; however, a lack of insight was significant among these participants. This lack of awareness of their diagnosis was evident when a participant reported that he had been diagnosed with malaria and had to keep the referral letter secret, only to be told later that he suffered from schizophrenia. This participant depended on the people he trusted and the health‐care professionals to be open and honest from the beginning about his condition; this planted in him a sense of doubt about the diagnosis of schizophrenia for more than 20 years. This is consistent with the finding that the attitude of health professionals towards the disclosure of a schizophrenia diagnosis has a significant impact on the acceptance of this diagnosis by mental health service users as well as their relatives.^34^ Literature indicates that a lack of insight can impede productivity, and the achievement of insight should be the first step towards rehabilitation.^35^ Sánchez and colleagues declared that the inability to accept disability was a significant barrier to individual productivity and social functioning.^36^

Additionally, this study revealed that individuals with schizophrenia have their own vision and expectations of life as well as the potential to live a meaningful life. Some of the participants dreamt of achieving their academic and career goals and even starting their own businesses; however, receiving suggestions for how their rehabilitation should proceed adversely affected their interests. This is consistent with a previous finding that a lack of interest results when an individual feels compelled to pursue a task, which serves as a significant constraint of the productivity of individuals with psychiatric conditions.^37^ Souraya and colleagues also revealed that giving schizophrenic individuals the liberty to make an informed decision was vital for goal‐orientated rehabilitation.^38^ A study also reported that these individuals do better in their career or rehabilitation activities if the choice was self‐initiated or self‐driven.^39^

Further, our study revealed barriers to the rehabilitation of people with schizophrenia. Resources such as housing affect the success of rehabilitation because even participants who can function independently within the community may have to remain accommodated in the hospital setting. Some of the study participants faced psychological implications due to their behaviours prior to diagnosis, leading to neglect from their family members. Such neglect impedes the success of rehabilitation. This is consistent with the finding that the public feels threatened by the presence of individuals with a history of schizophrenia coupled with violence.^40^ The participants in this study indicated a will to return home, but they feared rejection, being punished for their wrongdoing, or even having no house to go back to; therefore, in a country like Nigeria with poor resources, structured accommodation for such individuals should be considered within the community and at a reasonable price.

## 5 CONCLUSION

This study shows that the rehabilitation programme followed by the hospitals is a good initiative and has enabled the mental health service users to regain some level of independence and productive living. However, there are still lapses and weaknesses in the execution, such as variations in the approach to rehabilitation, the lack of a unified guide to rehabilitation, and prolonged institutionalization rather than re‐integration of the service users who lacked accommodation or acceptance by their relatives. Therefore, it can be concluded that the rehabilitation of individuals with schizophrenia, though a good effort, can be improved upon through active involvement of service users in their rehabilitation decision‐making process. This can potentially help mental health professionals better serve the interest of the service users and community as a whole.

## 6 RECOMMENDATIONS

Considering the demographic profile and the participants' reports of undergoing rehabilitation and learning a trade for many years, ranging from five to 30, there seems to be no end to rehabilitation. Therefore, the setting of broad, individualized goals before the commencement of rehabilitation is recommended. Further, collaborations between different settings and other parts of the world that have recorded evidence‐based success in rehabilitation are advisable. Wide variations were also noted between the intervention approaches taken by the health professionals; therefore, a practice guide for uniformity of practice is also recommended.

## ETHICAL APPROVAL

The full approval was secured from the first institution on the 19th October 2018 with approval number: NHREC 24/07/2013/PRO 12/18, and the second institution granted approval on the 30th October 2018 with approval number FNPH/HREC 18/10. Beneficence was promoted in this study, as there were no physical risks. Although the mental health service users are a vulnerable population, their rights were protected by strictly adhering to the enquiry on what this study is purposed. The inclusion criteria also considered mental health service users' vulnerability to determine those who were eligible to participate, part of which was clinical stability of 3 months, the evidence of which was confirmed through the follow‐up report. Co‐morbidity with an affective disorder that increases the tendency of the emotional breakdown was also excluded to minimize the emotional risk. This study posed no harm, although recounting the experience could bring about an emotional reaction in individuals with schizophrenia, despite the consideration of that for exclusion, the researcher, before the commencement of the research, liaised with the Psychology Unit of the hospitals that in the event any participant broke down emotionally during or even after the data collection would be referred at the researcher's cost; this was because mental health service users pay for this service and the section will be discontinued. The researcher developed a referral slip, and the Head of the Psychology Unit approved the referral of participants, but no participant had an emotional breakdown throughout the study.

## POSITIONING THE RESEARCHERS

Both researchers are Mental Health Lecturers. The first researcher is a Nigerian, a PhD Candidate and a registered psychiatric lecturer, lecturing in a university about 500km from the research settings, with no connection to the facility and the participants of the research settings. This allowed participants to express themselves freely. The researcher sought consent from each participant, and participant who declined participation was excused from the study without sanction or prejudice. The other researcher is a South African and a lecturer at a South African institution with no affiliation or any form of contact with the research settings.

## Data Availability

Data collected for this study and all documents on the research work were kept in a pass‐worded computer of the principal investigator, OO The data have no form of an identifier as pseudonyms were used to represent the participant's identity in compliance with the ethics requirement. The data dissemination statement was included, in consent (written and verbal) taken from the participants and in ethics application forms, that the data will be used for the research purpose and publications ONLY. Also in line with one of the requirements for securing ethical clearance for this study, data will only be divulged to individual readers on request with the consent of the institutional ethics committees where the study was conducted.

## References

[1] Loh SY . Interdisciplinary rehabilitation to facilitate recovery of people living with long‐term schizophrenia in developing countries. Psychotic Disorders: Update. 2018;63:63–78.

[2] Alphs L , Bossie C , Mao L , Lee E , Starr HL . Treatment effect with paliperidone palmitate compared with oral antipsychotics in patients with recent‐onset versus more chronic schizophrenia and a history of criminal justice system involvement. Early Intervent Psychiatry. 2018;12(1):55‐65. 10.1111/eip.12271 PMC5811784 26403322

[3] World Health Organization . The Global Burden of Disease, 2004 Update; 2004. www.who.int/healthinfo/global_burden_disease/GBD_report_2004update_full.pdf. Accessed March 16, 2018.

[4] World Health Organization . International Guidelines on Health‐related Rehabilitation; 2018. https://www.researchgate.net/…/273139344_WHO_International_Guidelines_on_Healthwww.who.int/medicines/areas/…/validation‐without_appendices_2016_05_17.pdf. Accessed March 16, 2018.

[5] World Health Organization . Schizophrenia; 2018. https://www.who.int/news‐room/fact‐sheets/detail/Schizophrenia. Accessed March 16, 2018.

[6] Kahn RS , Sommer IE . The neurobiology and treatment of first‐episode schizophrenia. Mol Psychiatry. 2015;20(1):84. 25048005 10.1038/mp.2014.66 PMC4320288

[7] Chong HY , Teoh SL , Wu DBC , Kotirum S , Chiou CF , Chaiyakunapruk N . Global economic burden of schizophrenia: a systematic review. Neuropsychiatr Dis Treat. 2016;12:357. 26937191 10.2147/NDT.S96649 PMC4762470

[8] Opoku‐Boateng YN , Kretchy IA , Aryeetey GC , et al. Economic cost and quality of life of family caregivers of schizophrenic patients attending psychiatric hospitals in Ghana. BMC Health Serv Res. 2017;17(2):697. 29219074 10.1186/s12913-017-2642-0 PMC5773918

[9] Wani RA , Dar MA , Margoob MA , Rather YH , Haq I , Shah MS . Diabetes mellitus and impaired glucose tolerance in patients with schizophrenia, before and after antipsychotic treatment. J Neurosci Rural Pract. 2015;6(1):17. 25552846 10.4103/0976-3147.143182 PMC4244781

[10] Kritharides L , Chow V , Lambert TJ . Cardiovascular disease in patients with schizophrenia. Med J Austr. 2017;207(4):179. 10.5694/mja17.00258 28814221

[11] Gordois AL , Toth PP , Quek RG , Proudfoot EM , Paoli CJ , Gandra SR . Productivity losses associated with cardiovascular disease: A systematic review. Expert Rev Pharmacoecon Outcomes Res. 2016;16(6):759‐769. 27831848 10.1080/14737167.2016.1259571

[12] Hird TR , Zomer E , Owen A , et al. The impact of diabetes on productivity in China. Diabetologia. 2019;62(7):1195‐1203. 31030220 10.1007/s00125-019-4875-4

[13] Thonse U , Behere RV , Frommann N , Sharma P . Social cognition intervention in schizophrenia: description of the training of affect recognition program–Indian version. Asian J Psychiatry. 2018;31:36‐40. 10.1016/j.ajp.2017.12.015 29358102

[14] Bejerholm U , Roe D . Personal recovery within positive psychiatry. Nord J Psychiatry. 2018;72(6):420‐430. 30383472 10.1080/08039488.2018.1492015

[15] Slade M . Implementing shared decision making in routine mental healthcare. World Psychiatry. 2017;16(2):146‐153. 28498575 10.1002/wps.20412 PMC5428178

[16] Morant N , Kaminskiy E , Ramon S . Shared decision making for psychiatric medication management: beyond the micro‐social. Health Expect. 2016;19(5):1002‐1014. 26260361 10.1111/hex.12392 PMC5053275

[17] Phelps K , Hodgson J , Heru A , Jensen J . Medical family therapy in psychiatry. In: Clinical Methods in Medical Family Therapy. Cham: Springer; 2018:231‐259. 10.1007/978-3-319-68834-3

[18] Hill H , Killaspy H , Ramachandran P , Ng RMK , Bulman N , Harvey C . A structured review of psychiatric rehabilitation for individuals living with severe mental illness within three regions of the Asia‐Pacific: implsications for practice and policy. Asia‐Pacific Psychiatry. 2019;11(2):e12349. 30734499 10.1111/appy.12349

[19] Oren R , Orkibi H , Elefant C , Salomon‐Gimmon M . Arts‐based psychiatric rehabilitation programs in the community: perceptions of healthcare professionals. Psychiatr Rehabil J. 2019;42(1):41. 30221966 10.1037/prj0000325

[20] Ahmed AO , Marino BA , Rosenthal E , et al. Recovery in schizophrenia: what consumers know and do not know. Psychiatric Clin North Am.. 2016;39(2):313‐330. 10.1016/j.psc.2016.01.009 27216905

[21] Kukla M , Whitesel F , Lysaker PH . An integrative psychotherapy approach to foster community engagement and rehabilitation in schizophrenia: a case study illustration. J Clin Psychol. 2016;72(2):152‐163. 26636563 10.1002/jclp.22248

[22] Thara R , Kamath S . Women and schizophrenia. Indian J Psychiatry. 2015;57(6):246. 10.4103/0019-5545.161487 PMC4539869 26330642

[23] Lucksted A , Drapalski AL , Brown CH , et al. Outcomes of a psychoeducational intervention to reduce internalized stigma among psychosocial rehabilitation clients. Psychiatric Serv. 2016;68(4):360‐367. 10.1176/appi.ps.201600037 27903136

[24] Gürhan N , Beşer NG , Polat Ü , Koç M . Suicide risk and depression in individuals with chronic illness. Community Ment Health J. 2019;55(5):840‐848. 30848413 10.1007/s10597-019-00388-7

[25] Brooke‐Sumner C , Petersen I , Asher L , Mall S , Egbe CO , Lund C . Systematic review of feasibility and acceptability of psychosocial interventions for schizophrenia in low and middle income countries. BMC Psychiatry. 2015;15(1):19. 25886524 10.1186/s12888-015-0400-6 PMC4382830

[26] Charlson FJ , Ferrari AJ , Santomauro DF , et al. Global epidemiology and burden of schizophrenia: findings from the global burden of disease study 2016. Schizophr Bull. 2018;44(6):1195‐1203. 29762765 10.1093/schbul/sby058 PMC6192504

[27] Heaton MM . The politics and practice of Thomas Adeoye Lambo: towards a post‐colonial history of transcultural psychiatry. Hist Psychiatry. 2018;29(3):315‐330. 29582688 10.1177/0957154X18765422

[28] Rossi KC , Kim AM , Jetté N , Yoo JY , Hung K , Dhamoon MS . Increased risk of hospital admission for ICD‐9‐CM psychotic episodes following admission for epilepsy. Epilepsia. 2018;59(8):1603‐1611. 29974458 10.1111/epi.14508

[29] Krippendorff K . Content Analysis: An Introduction to Its Methodology. Sage Publications; 2018. http://repository.upenn.edu/asc_papers/226

[30] Evensen S , Wisløff T , Lystad JU , et al. Exploring the potential cost‐effectiveness of a vocational rehabilitation program for individuals with schizophrenia in a high‐income welfare society. BMC Psychiatry. 2019;19(1):140. 31064371 10.1186/s12888-019-2130-7 PMC6505225

[31] Askari M . A comparative study of physical activity level among in‐service users with schizophrenia and bipolar disorder. Int Arch Health Sci. 2018;5(3):93.

[32] Hasan S , Adil M . Schizophrenia: a neglected problem in Pakistan. The Lancet. 2019;394(10193):115‐116. 10.1016/S0140-6736(19)30290-9 31305248

[33] Moorkath F , Vranda MN , Naveenkumar C . Women with mental illness–an overview of sociocultural factors influencing family rejection and subsequent institutionalization in India. Indian J Psychol Med. 2019;41(4):306. 31391661 10.4103/IJPSYM.IJPSYM_123_19 PMC6657485

[34] Amsalem D , Hasson‐Ohayon I , Gothelf D , Roe D . How do service users with schizophrenia and their families learn about the diagnosis? Psychiatry. 2018;81(3):283‐287. 30020033 10.1080/00332747.2018.1443676

[35] De Jong S , Hasson‐Ohayon I , van Donkersgoed RJ , et al. Predicting therapy success from the outset: the moderating effect of insight into the illness on metacognitive psychotherapy outcome among persons with schizophrenia. Clin Psychol Psychother. 2019;26(6):650‐660. 31270887 10.1002/cpp.2388

[36] Sánchez J , Sung C , Phillips BN , et al. Predictors of perceived social effectiveness of individuals with serious mental illness. Psychiatr Rehabil J. 2019;42(1):88. 30265067 10.1037/prj0000321

[37] Corbière M , Zaniboni S , Dewa CS , et al. Work productivity of people with a psychiatric disability working in social firms. Work (Preprint). 2019;62(1):151‐160. 10.3233/WOR-182850 30689598

[38] Souraya S , Hanlon C , Asher L . Involvement of people with schizophrenia in decision‐making in rural Ethiopia: a qualitative study. Glob Health. 2018;14(1):85. 10.1186/s12992-018-0403-4 PMC6103856 30134989

[39] Asher L , Hanlon C , Birhane R , et al. Community‐based rehabilitation intervention for people with schizophrenia in Ethiopia (RISE): a 12‐month mixed‐methods pilot study. BMC Psychiatry. 2018;18(1):250. 30075715 10.1186/s12888-018-1818-4 PMC6091097

[40] Chen X , Zhang X , Wong SC , Yang M , Kong D , Hu J . Characteristics of alleged homicide offenders with and without schizophrenia in Sichuan, China. Crim Behav Mental Health. 2018;28(2):202‐215. 10.1002/cbm.2054 29052283

